# Incorporating Wastewater Sludge as a Cement Alternative in Repair Mortar: An Experimental Study of Material Properties

**DOI:** 10.3390/ma17225625

**Published:** 2024-11-18

**Authors:** Jeong-Bae Lee

**Affiliations:** Department of Smart Construction and Environmental Engineering, Daejin University, 1007 Hoguk-ro, Pocheon-si 11159, Republic of Korea; jblee@daejin.ac.kr

**Keywords:** waste water sludge, repair mortar, pretreatment, additive material, substitution

## Abstract

The global construction industry faces increasing pressure to adopt sustainable practices, particularly in reducing cement-related CO_2_ emissions. This study investigates the feasibility of using treated wastewater sludge (WWS) as a partial replacement for cement in repair mortars. Treated (A-WWS) and untreated (B-WWS) sludge were evaluated for their effects on workability, mechanical strength, durability, and environmental impact. Flow tests revealed that A-WWS maintained workability similar to the control mixture, while B-WWS reduced flow due to its coarser particles. Compressive strength tests showed that a 10% A-WWS substitution improved strength due to enhanced pozzolanic reactions, while untreated sludge reduced overall strength. Water absorption and bond strength tests confirmed the improved durability of A-WWS mortars. Chemical attack resistance testing demonstrated that A-WWS significantly reduced carbonation depth and chloride penetration, enhancing durability. Microstructural analysis supported these findings, showing denser hydration products in pretreated sludge mixtures. An environmental hazard analysis confirmed low heavy metal content, making sludge-based mortars environmentally safe. Although wastewater sludge shows promise as a partial cement replacement, the processing energy demand remains substantial, necessitating further investigation into energy-efficient treatment methods. This research highlights the potential of pretreated WWS as a sustainable alternative in construction, contributing to reduced cement consumption and environmental impact without compromising material performance. The findings support the viability of sludge-based repair mortars for practical applications in the construction industry.

## 1. Introduction

The global construction industry is currently undergoing a significant shift, driven by an increasing focus on sustainability and environmental responsibility. One of the key challenges facing this industry is the enormous consumption of natural resources, particularly cement, which is a major contributor to environmental degradation [1]. Cement production is responsible for approximately 8% of global anthropogenic CO_2_ emissions, making it one of the largest industrial contributors to climate change [2]. As a result, there has been a growing push toward finding sustainable alternatives to traditional construction materials in an effort to reduce the industry’s environmental impact. This has led to an exploration of waste materials as potential substitutes for conventional resources. Among these alternatives, wastewater sludge has emerged as a promising candidate due to its availability and potential for use as a partial cement replacement [3,4,5].

Wastewater sludge (WWS), a byproduct of wastewater treatment plants (WWTPs), presents significant environmental and economic challenges due to the large quantities produced and the limited sustainable disposal options available. Composed primarily of organic matter, suspended solids, and trace amounts of heavy metals, wastewater sludge is typically disposed of through methods such as landfilling, incineration, or agricultural application [6]. However, these methods are becoming increasingly unsustainable due to rising disposal costs and the tightening of environmental regulations. Consequently, researchers have been seeking innovative ways to transform wastewater sludge from a waste product into a valuable resource, particularly in the construction industry [7,8,9]. One of the most compelling reasons for considering WWS as a cement alternative lies in its chemical composition. With appropriate treatment, the sludge can be processed into a material that exhibits pozzolanic properties—meaning it can react with calcium hydroxide in the presence of water to form compounds that contribute to the hardening of mortar, similar to the function of cement [10,11,12]. This could potentially reduce the demand for raw materials traditionally used in cement production, such as limestone and clay, and decrease the overall carbon footprint of the construction process. Moreover, the utilization of wastewater sludge addresses two significant environmental concerns: reducing the waste burden on disposal systems and minimizing the CO_2_ emissions associated with cement production [13,14]. Recent research has highlighted various types of sludge and other waste materials, such as fly ash, blast furnace slag, and agricultural residues, as potential components in eco-friendly construction materials. Studies have shown that incorporating these materials in concrete and mortar not only improves the sustainability profile of construction projects but also helps mitigate waste disposal issues by diverting significant quantities of waste from landfills [15,16,17]. For example, fly ash and blast furnace slag have been widely used as supplementary cementitious materials, offering improved durability and reducing the overall cement requirement in concrete. Similarly, studies on agricultural wastes like rice husk ash and sugarcane bagasse ash have demonstrated their pozzolanic properties, which enhance the mechanical strength and durability of cement-based products while reducing their environmental impact. In addition to wastewater sludge, municipal sewage sludge has been investigated as a sustainable binder component in concrete. Research indicates that with adequate pre-treatment, such as thermal or chemical activation, municipal sewage sludge can develop pozzolanic characteristics, contributing to improved binding properties in concrete mixtures [12,18,19]. Researchers have also explored the use of paper mill sludge and textile sludge, both of which contain substantial amounts of organic and inorganic compounds that, after processing, can improve the workability and strength of construction materials.

Repair mortar is an essential material in construction, playing a critical role in the restoration and maintenance of concrete structures. With the global infrastructure aging, there is a growing demand for repair materials that are not only durable but also environmentally friendly. Repair mortars typically consist of a mixture of cement, sand, water, and additives, designed to restore the structural integrity of concrete while providing adequate strength and durability [20]. However, the use of cement in these mixtures continues to raise environmental concerns due to the associated energy consumption and carbon emissions during its production. By incorporating WWS as a partial cement replacement in repair mortar, there is an opportunity to produce a more sustainable product without compromising on the essential properties required for effective structural repair [20,21,22]. The incorporation of WWS as additives as partial replacements for Portland cement has become increasingly important, not only to mitigate the environmental impact of cement production but also to enhance the durability of concrete structures subjected to aggressive conditions, such as sulfate attack, chloride exposure, and alkali–aggregate reactions [12,23,24,25]. The use of sludge-based repair mortar has the potential to reduce the demand for virgin materials in construction, thereby promoting resource efficiency and environmental sustainability.

The environmental and economic benefits of using sludge and waste materials in construction extend beyond CO_2_ reduction. For instance, waste-derived binders can improve the durability of concrete in harsh environments by enhancing its resistance to sulfate attack, chloride penetration, and freeze–thaw cycles, crucial for infrastructure longevity [24]. By reducing the need for virgin materials, these alternative binders also alleviate the pressure on natural resources and contribute to a circular economy within the construction sector. The sludge’s chemical composition, influenced by factors such as the source of the wastewater and the treatment processes it undergoes, can have a significant impact on the mortar’s behavior. As such, it is necessary to develop suitable processing techniques to treat the sludge, removing contaminants and enhancing its reactivity. This could involve drying, incineration, or chemical stabilization to improve its pozzolanic properties [26,27].

The objectives of this research are to investigate the feasibility of using treated wastewater sludge as a partial replacement for cement in repair mortars and to assess the material properties that determine its practical applicability in construction. By examining various treatment methods and replacement ratios, the study aims to identify the optimal conditions under which sludge-based mortars can achieve performance comparable to conventional repair mortars while offering environmental benefits. A thorough evaluation of the mechanical and durability properties of these mortars will help establish their viability for real-world applications, ensuring that they can withstand the demands of structural repairs.

Through adherence to standard specifications and rigorous testing protocols, including assessments of compressive and flexural strength, water absorption, and durability, this study reveals the potential of WWS as a viable alternative to traditional cement in mortar fabrication. By conducting a comprehensive investigation into the physio-mechanical properties of the resulting mortar, this research offers valuable insights into the performance of sludge-based mortars, ensuring they meet the necessary criteria for practical application in structural repairs. Furthermore, this research emphasizes the importance of conducting an environmental hazard analysis to fully understand the sustainability implications of incorporating wastewater sludge into construction materials.

## 2. Experimental Procedure

### 2.1. Raw Materials

In this experimental investigation, ordinary Portland cement (OPC) was used as the primary binding agent. The chemical and physical properties of OPC are summarized in Table 1, following the standards specified in ASTM C150 [28]. For the fine aggregates, natural sand with a maximum particle size of 4.75 mm was used, conforming to the grading requirements outlined in ASTM C33-2003 [29]. This ensured that the aggregates met the necessary standards for use in concrete mixes. A key component of this study was the use of wastewater treatment plant sludge (WWS), both in untreated and treated forms, as a supplementary cementitious material (SCM). The pretreatment of WWS aimed to enhance its pozzolanic reactivity and improve particle compatibility with the cement matrix. To achieve this, the sludge was subjected to a controlled ball milling process, performed at 300 rpm for 2 h, using steel balls as grinding media to achieve a targeted particle size reduction. The milling process was carefully monitored to ensure consistent energy input, which was found to be crucial for achieving the optimal particle fineness and surface area required to activate pozzolanic properties.

**Figure 1:**
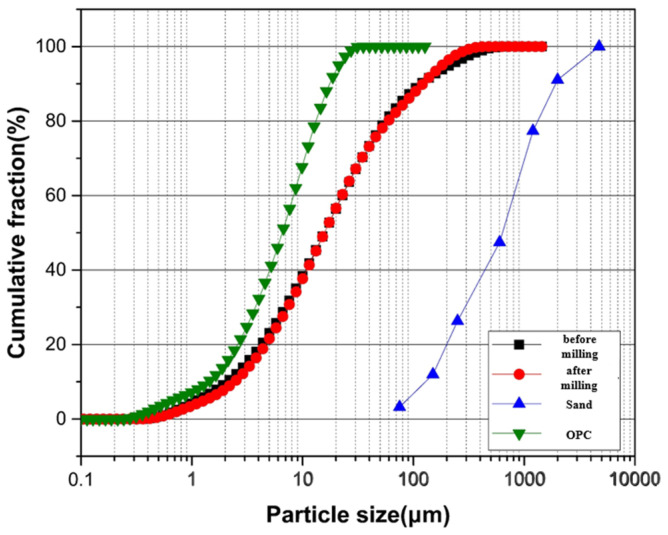
Particle size distribution of material used.

**Figure 2:**
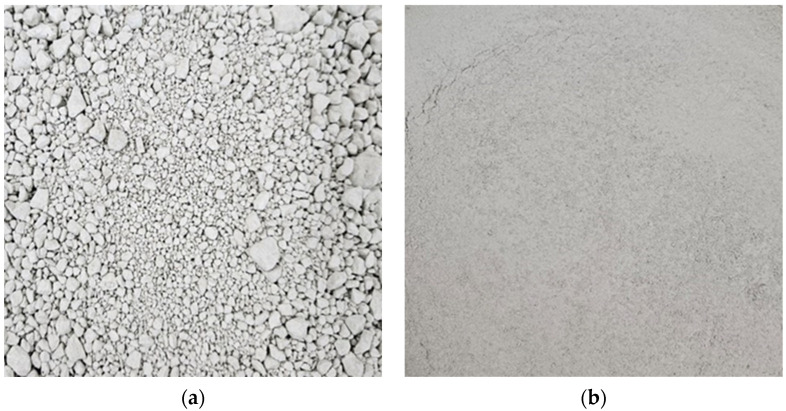
(**a**) Before and (**b**) after milling of WWTS.

The milling process improved the sludge’s homogeneity, eliminated larger agglomerates, and produced a fine, uniform powder with a high specific surface area. Following pretreatment, a comprehensive analysis of the sludge’s chemical composition, mineralogical content, and physical properties including specific gravity, surface area, and particle size distribution was conducted to thoroughly understand its impact on the mortar’s performance. The results of these analyses are detailed in Table 2, and visual representations are provided in Figure 1 and Figure 2, respectively. To improve the workability of the cement mortar, a polycarboxylic-based water-reducing admixture was incorporated into the mix. The inclusion of this admixture was crucial for maintaining a consistent flow and enhancing the overall performance of the fresh mortar. The physical and chemical properties of the water reducer are listed in Table 3. This thorough selection and preparation of raw materials ensured that the experimental conditions were both standardized and suitable for evaluating the performance of the mortar mixes under various testing conditions.

### 2.2. Mix Proportion and Specimen Preparation

In this study, five sets of mortar specimens were prepared, each with distinct mix proportions, as detailed in Table 4, utilizing wastewater treatment plant sludge (WWS) as a supplementary cementitious material (SCM). Cement was partially replaced at levels of 10% and 20%, chosen based on preliminary pilot testing and previous literature findings. These levels were selected to assess the effects of moderate (10%) and higher (20%) substitution, balancing environmental benefits with the mechanical performance of the mortar. Higher substitution levels, while possible, often result in a decrease in mechanical strength due to the lower reactivity of sludge as compared to cement. Thus, 10% and 20% were chosen as feasible initial ratios, with future studies planned to investigate additional substitution ratios to refine these parameters further. A consistent water-to-cement ratio of 0.4 was maintained throughout the experiments. To improve the workability of the fresh mortar, a yellow-colored polycarboxylic water-reducing admixture was added at a dosage of 0.7% of the total mix [19,30]. The preparation process began with dry mixing the cement and aggregate for 3 min to ensure an even distribution of the dry components. Afterward, water and the water-reducing agent were gradually added to the mixture, followed by an additional 5 min of mixing to achieve a uniform consistency. The freshly prepared mortar was then carefully compacted into molds by tamping, ensuring proper compaction and minimizing air voids. After 24 h of air curing, the specimens were demolded and immersed in water at a controlled temperature of 23.2 °C for 28 days, facilitating optimal hydration and curing. Specimens for each mix proportion were prepared in accordance with Table 3, with specific geometries selected depending on the type of test being conducted. Flow and setting time tests were performed on the fresh mortar mixtures immediately after preparation. For microstructural analysis, such as X-ray diffraction (XRD), and environmental hazard assessment, powder samples with particle sizes below 75 µm were obtained by grinding the specimens and using standard sieve sizing methods. These powder samples were extracted from the crushed specimens used in the mechanical strength tests. For scanning electron microscopy (SEM) analysis, small samples approximately 0.5 cm in size were meticulously extracted from the inner core of the specimens to ensure an accurate representation of the microstructure. To prevent unwanted hydration before SEM testing, these samples were stored in isopropanol. This detailed and careful preparation of specimens ensured accurate and reliable results across all tests, providing critical insights into the performance characteristics of the mortar mixtures.

### 2.3. Experimental Methods and Methodology

#### 2.3.1. Flow Tests and Setting Time

**Figure 3:**
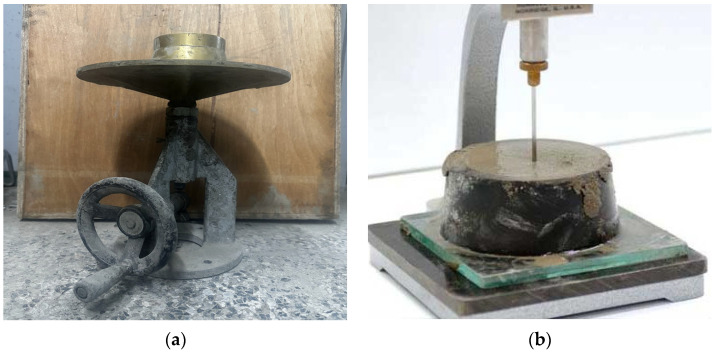
(**a**) Followability test and (**b**) setting time test of WWTS mortar.

The flow test was conducted in accordance with ASTM C 1437 standards [31], using a standard flow mold with upper and lower open diameters of 70 and 100 mm, respectively, and a height of 50 mm. The mold was carefully positioned at the center of the flow table. To start the test, a 25 mm-thick layer of mortar was compacted by applying 20 consistent tamps. A second layer was then added until the mold slightly overflowed, after which the surface was leveled, and the mold was removed. The flow table was then dropped 25 times within a 15-s period. The diameter of the fresh mortar was measured along four predetermined guidelines, and the average was calculated to determine the flow spread diameter. Additionally, the workability of the repair cement mortar was assessed by measuring its setting time. This was conducted following the procedures outlined in relevant literature. Figure 3 shows the complete testing setup, including the apparatus for both flow and setting time tests. These tests are essential for understanding the performance characteristics of the mortar, ensuring its suitability for repair applications.

#### 2.3.2. Mechanical Strength Test

An extensive experimental study was conducted to evaluate the mechanical strength of both cubic and prismatic specimens, allowing for a comprehensive assessment of the material’s performance under different stress conditions. For compressive strength tests, cubic specimens with dimensions of 50 × 50 × 50 mm^3^ were prepared and tested before and after applying electric stress. These tests were conducted using a Shimadzu CCM-200A universal testing machine (Shimadzu, CCM-200A, Shimadzu Corporation, Kyoto, Japan) with a capacity of 200 tons, following the ASTM C109 standards [32], to ensure the consistency and comparability of results. To determine the flexural strength, prismatic specimens measuring 40 × 40 × 160 mm^3^ were tested using a Shimadzu AG-I 250 KN flexural bending machine (Shimadzu, AG-I 250 KN, Shimadzu Corporation, Kyoto, Japan) with a 25-ton capacity, in accordance with ASTM C348 guidelines [33]. This setup provided a controlled environment to measure the bending strength of the material, which is crucial for applications where resistance to cracking or deformation is needed. For each mix proportion, five specimens were prepared and tested to ensure the reliability and reproducibility of the results. The average strength value was then calculated based on these five specimens, giving a representative measure of the material’s mechanical properties. This approach minimized the impact of any anomalies or inconsistencies in individual samples, providing a more accurate and reliable assessment of the material’s overall strength performance.

#### 2.3.3. Water Absorption Rate Test

The water absorption rate test was conducted in accordance with ASTM C642-21 standards [34], which define water absorption as a material’s ability to absorb and retain water. This test involved measuring the water saturation level of each test specimen. The samples were first dried in an oven at a temperature of 105 °C until they reached a constant weight (m_1_). Following this, the specimens were fully submerged in water at (20 ± 2) °C, with the water level maintained 50 mm above the top surface of the sample. At 24-h intervals, each sample was removed from the water, briefly air-dried to remove excess surface water, and weighed with an accuracy of 0.1% (m_2_). The saturation of the samples was considered complete when the difference between consecutive weighings did not exceed 0.1%. The water absorption (Wabs) was then calculated based on the increase in weight relative to the dry sample weight. This test is essential for understanding the material’s behavior in wet environments, as the capacity to absorb and retain water significantly affects its durability and performance over time in real-world applications.

(1) $$W_{abs}=\frac{m_{2}-m_{1}}{m_{1}}\cdot 100\%$$

where W_ebs_ is the water absorption in percentage, m_2_ is the mass of the test sample after saturation, and m_1_ is the mass of the air-dried test sample.

#### 2.3.4. Chloride Penetration Test

**Figure 4:**
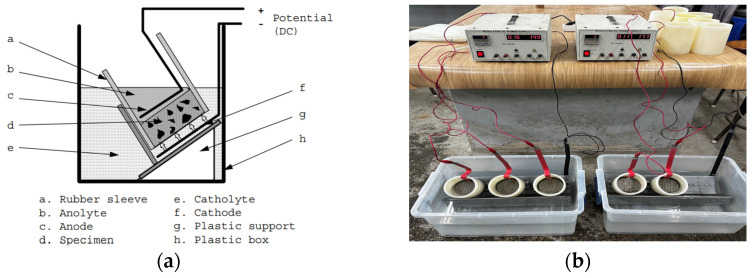
Chloride penetration test: (**a**) NT-Build 492 standard and (**b**) actual experimental setup.

The chloride penetration test was conducted in accordance with NT-BUILD 492 standards [35], as illustrated in Figure 4. Cylindrical specimens with a height of 50 mm and a diameter of 100 mm were used to assess the material’s resistance to chloride ingress. Before testing, the specimens were conditioned by immersing them in a calcium hydroxide (Ca(OH)_2_) solution for 18 h to stabilize the material and ensure uniform saturation. The test was carried out over a 24-h period under controlled temperature and humidity conditions, during which a voltage of 30 V and a current of 0.46 mA were applied to the water-washed (WWS) specimens. Following this period, the specimens were air-dried for one day to prepare them for analysis. Each specimen was then split hydraulically to expose a fresh cross-section. To determine the depth of chloride penetration, a silver nitrate (AgNO_3_) solution was applied to the freshly split surface, which reacts with the chlorides to create a visible indication of penetration depth. A 10 mm section on both ends of the specimen was excluded from measurement to minimize potential edge effects that could affect accuracy. The chloride penetration length was then measured at seven points, spaced 10 mm apart along the smoother surface of the specimen, and the average depth was calculated. This test is essential for evaluating the material’s resistance to chloride ingress, a key factor in predicting its durability, especially in environments exposed to de-icing salts or marine conditions where chloride penetration can accelerate degradation.

#### 2.3.5. Carbonation Depth Test

**Figure 5:**
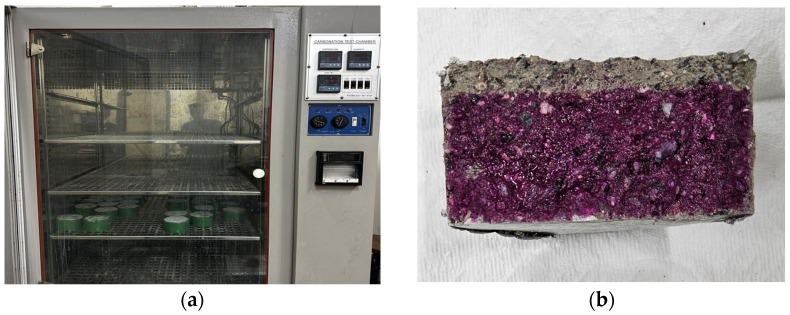
(**a**) Accelerated carbonation chamber and (**b**) carbonation depth measurement.

Specimens for the carbonation depth test were prepared in accordance with ISO 1920-12:2015 standards [36]. After 24 h of casting, 120 specimens were demolded for each concrete grade and cured for 28 days in a controlled environment of (20 ± 1) °C and (95 ± 5)% relative humidity. Following this curing period, it takes hours to remove any residual moisture. Then, the specimens are placed in an environmental simulation test chamber for one week to stabilize their temperature and humidity with the surrounding environment as shown in Figure 5a,b. To accelerate carbonation testing, a high CO_2_ concentration was applied, utilizing an accelerated carbonation method facilitated by the environmental simulation system. The carbonation depth was assessed by applying a 1% phenolphthalein solution in 95% ethyl alcohol to the specimen’s cross-section. This reagent visibly marks the depth of carbonation, as it changes color upon contact with non-carbonated areas. Ten points on the specimen’s cross-section were measured for carbonation depth, with an accuracy of 0.1 mm, and their average value was determined. This test provides insight into the material’s resistance to carbonation, an important factor in evaluating the durability of concrete exposed to atmospheric CO_2_ over time.

#### 2.3.6. Freeze–Thaw Test

In this experimental study, the freeze–thaw test was conducted to evaluate the durability of repair materials containing WWTPs under cyclic thermal stress. The thermal cycling protocol required that the core temperature of the specimens be maintained within a range of −18 ± 2 °C to 6 ± 2 °C for a period of 4 h per cycle. This cycle was repeated 300 times to simulate prolonged exposure to freezing and thawing conditions. To assess the material’s durability, the relative dynamic modulus (RDM) and mass loss were measured at intervals of every 30 cycles.

(2) $$P_{r}=\frac{n_{m}^{2}}{n_{0}^{2}}\times 100$$

The procedure followed the guidelines set forth by ASTM [37], ensuring consistency and accuracy. The RDM was calculated using Equation (2), where Pr represents the relative dynamic modulus (%), n_m_ denotes the measured frequency after m cycles of Fourier transform (FT), and n_0_ signifies the estimated frequency at the initiation of FT cycling. These measurements are crucial for understanding how the material behaves under repeated freeze–thaw conditions and for predicting its long-term performance in climates with fluctuating temperatures.

#### 2.3.7. Microstructure Analysis

The microstructural properties of both the cement mortar and conductive mortar were investigated using advanced analytical techniques. X-ray diffraction (XRD) was employed to analyze the crystallographic structure of the materials. This analysis was conducted using a Goniometer Ultima+ instrument from Mitsubishi Tanabe Pharma, Osaka, Japan. The XRD measurements were performed with a wavelength of 1.54 Å, scanning over a 2θ range of 10°–65° at a scanning speed of 2°/min. The instrument operated with an applied voltage of 40 kV and a current of 30 mA, ensuring precise and accurate results. This technique enabled the identification of various crystalline phases present in both the cement and conductive mortar, providing insights into their structural composition.

Furthermore, scanning electron microscopy (SEM) analysis was conducted to investigate the compositional and morphological characteristics of the wastewater treatment plant (WWTP) particles incorporated within the mortar matrix. The SEM technique was selected due to its ability to generate high-resolution images of the material’s microstructure, allowing for a detailed examination of both particle dispersion and morphological integration on the sub-micron scale. During the SEM analysis, secondary electron imaging was utilized to highlight surface topography, while backscattered electron imaging provided contrast based on atomic number, which is instrumental in distinguishing between the WWTP particles and the surrounding matrix.

Through SEM, it was observed that the WWTP particles exhibited a heterogeneous distribution within the cement matrix, with certain regions showing clusters of particles that could influence the mortar’s structural properties. The analysis revealed surface morphology details such as particle roughness and porosity, which are essential for understanding the potential bonding interactions between the WWTP particles and the cementitious material. The distribution pattern and degree of particle-matrix adhesion were also carefully assessed, as these factors play a critical role in the overall durability, mechanical strength, and porosity of the mortar.

The SEM analysis followed methodologies established in previous studies [38], allowing for consistent and reliable results. Insights from SEM imaging suggested that the presence of WWTP particles introduced unique interactions within the mortar matrix, potentially enhancing properties such as crack resistance and load distribution. Moreover, the particle–matrix interface was observed to vary depending on the concentration and size distribution of the WWTP particles, with larger particles appearing to contribute more significantly to mechanical reinforcement. These findings provided valuable information on how WWTP particles could be optimized to improve the structural performance of the mortar, supporting further exploration of eco-friendly construction materials incorporating recycled components.

#### 2.3.8. Environmental Hazard Analysis

In this experimental investigation, it was crucial to assess the environmental impact of WWTPs, a waste by-product generated in the treatment plant. To determine the presence of potentially hazardous substances, an environmental hazard analysis was conducted using inductively coupled plasma mass spectrometry (ICP-MS). This technique, as discussed in the previous studies [39], allows for the precise detection of ions within a sample by utilizing inductively coupled plasma to ionize the sample material. ICP-MS is highly sensitive, with a detection limit in the parts per billion (ppb) range, making it an ideal tool for both qualitative and quantitative analysis of trace elements. By using this method, the experiment was able to simultaneously detect and measure even minute concentrations of hazardous substances that might pose environmental risks. The analysis provided critical data on the presence of any contaminants within the wheel wash sludge, contributing to a comprehensive evaluation of its suitability for use as a supplementary cementitious material, while also ensuring that its use aligns with environmental safety standards.

## 3. Results and Discussion

### 3.1. Flow Tests and Setting Time

**Figure 6:**
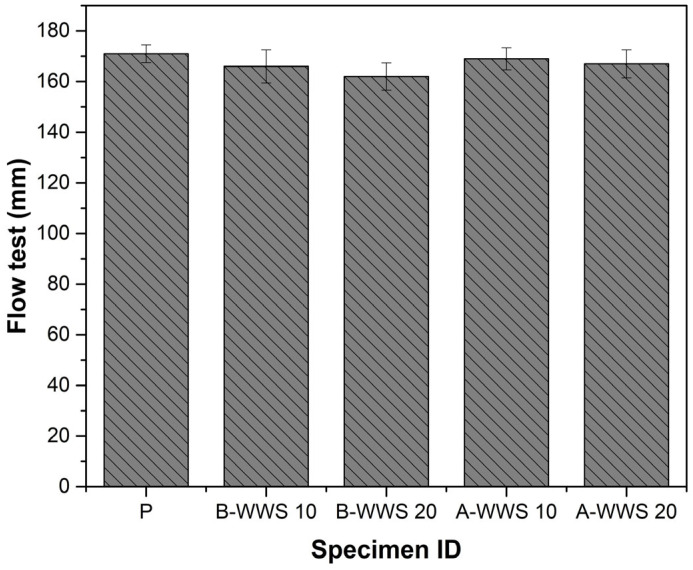
Flow test of mortar.

The effect of both treated (A-WWS) and untreated (B-WWS) wastewater sludge on the workability of concrete was assessed using flow tests, as illustrated in Figure 6. The results suggest a nuanced impact on flow properties, depending on the treatment type and substitution level. Overall, the substitution of cement with both B-WWS and A-WWS showed changes in workability, which were influenced by the characteristics of the sludge and its interaction with other constituents. The results indicate a slight reduction in flow with increasing levels of B-WWS, suggesting that the untreated sludge might contribute to increased friction between aggregate particles, thereby slightly reducing the fresh mix’s mobility. Specifically, B-WWS at 10% and 20% substitution levels displayed reductions in flow when compared to the plain mix (P). This decrease is attributed to the presence of untreated, coarser particles, which tend to absorb more water and interfere with the smooth movement of the fresh mixture [40]. In contrast, substituting cement with A-WWS (after ball milling) did not significantly alter flow characteristics relative to the plain mix (P). The ball milling process likely improved particle size uniformity and reduced surface roughness, enabling better particle packing and reducing internal friction within the mix. This helped maintain a level of workability comparable to the control specimen [41]. The mechanical grinding of A-WWS appears to mitigate the negative effects on flowability seen in the untreated B-WWS by producing finer particles with higher specific surface areas, which can improve the distribution of the cementitious material and enhance the overall lubrication effect.

The consistency in A-WWS mixtures may also result from improved dispersion facilitated by ball milling. The milling process likely reduced larger, flocculated sludge particles into smaller, more uniform sizes, enhancing compatibility with the superplasticizer [8]. The superplasticizer’s steric repulsion effect was maintained more effectively in A-WWS mixtures, preventing particle agglomeration and promoting an improved slump performance compared to untreated WWS. The observed trend in flow behavior can be associated with a balance between paste volume and water absorption [7]. A-WWS, with significant size reduction, presents less obstruction to aggregate movement, whereas B-WWS’s larger particles may hinder free movement and increase friction. The results indicate that at higher substitution levels (10%), the effects of sludge treatment are more pronounced, underscoring the importance of particle modification in preserving workability.

**Figure 7:**
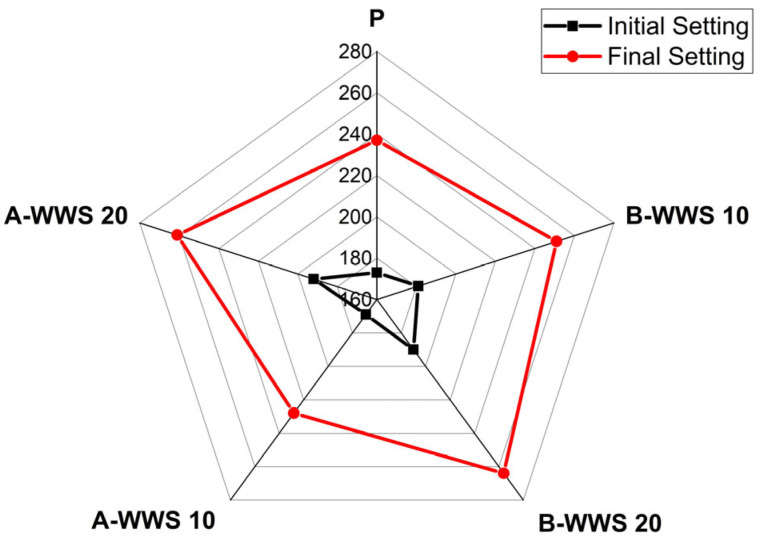
Setting time.

The results depicted in Figure 7 demonstrate that the substitution of cement with WWS significantly delays both initial and final setting times, with a more pronounced extension observed at higher levels of cement substitution. This trend suggests that WWS, particularly in higher amounts, has a retarding effect on the hydration process, likely due to its influence on key chemical reactions [12]. The prolonged setting time is attributed to a reduced rate of ettringite formation from gypsum and water reactions, as well as slower hydration involving alite and belite. There is also a notable contrast between treated and untreated sludge effects on setting times. Untreated B-WWS, used at both 10% and 20% substitution levels, led to significant delays in both initial and final setting times, likely due to its coarser particle size, which reduces reactive site availability and slows hydration overall. Additionally, the lower reactivity of untreated WWS can impede portlandite formation, further delaying pozzolanic reactions that contribute to the setting process [42]. Conversely, the use of treated A-WWS (after ball milling) exhibited a slight reduction in setting time compared to its untreated counterpart. The ball milling process likely enhanced the reactivity of the sludge by producing finer particles with increased surface area, thereby facilitating a more efficient reaction with calcium hydroxide during hydration. This increased reactivity contributes to a more rapid formation of the C-S-H gel, ultimately reducing the setting time compared to B-WWS. The reduction in setting time with A-WWS can be partially attributed to the better dispersion of particles, which allows for more effective hydration and gel formation, thus accelerating the setting process [15].

### 3.2. Mechanical Strength

**Figure 8:**
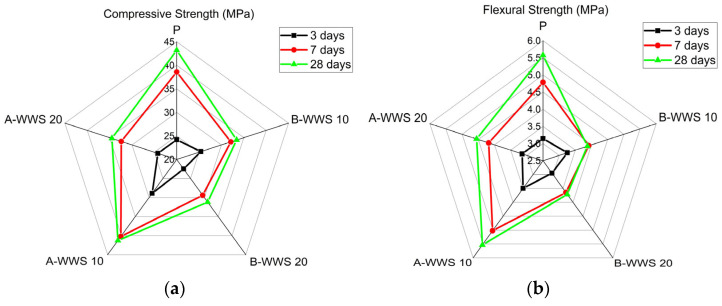
Mechanical property (**a**) compressive strength and (**b**) flexural strength.

The compressive strength characteristics of WWS before and after pretreatment were analyzed through a series of compressive strength tests in this experimental study. Figure 8a presents the results of these tests for samples containing WWS. The compressive strength test results revealed that the general sample exhibited strengths of 24.2 and 38.6 MPa after 3 and 7 days, respectively, and 43.2 MPa after 28 days. In contrast, the sample with a 10% replacement of untreated wastewater treatment facility sludge showed a compressive strength of 25.4 MPa after 3 days, followed by 32.1 MPa after 7 days and 33.4 MPa after 28 days. Although the initial strength of the 10% replacement sample was slightly higher than that of the general sample, the strength after 7 and 28 days demonstrated a decreasing trend. Similarly, the 20% replacement sample exhibited a decline in strength across all time intervals (3, 7, and 28 days). This reduction in compressive strength with higher WWS content can be attributed to the lower reactivity of untreated WWS. Untreated WWS lacks the necessary binding properties, such as the generation of calcium hydroxide (Ca(OH)_2_) during hydration, which aids in the pozzolanic effect—where reactive silica in the sludge reacts with Ca(OH)_2_ to form additional calcium silicate hydrates (C-S-H) that contribute to strength development [43]. Without proper pretreatment, the WWS particles tend to act more as fillers than reactive components.

For the samples where the WWS underwent a pretreatment process, the 10% replacement sample showed improved strength values of 28.9 and 40.2 MPa after 3 and 7 days, respectively, with a final strength of 41.3 MPa after 28 days. This improvement in strength after pretreatment could be attributed to the thermal decomposition of calcium carbonate (CaCO_3_) into calcium oxide (CaO), which enhances reactivity [44]. CaO reacts with water to form Ca(OH)_2_, supporting the pozzolanic reaction, which enhances the formation of C-S-H, contributing to overall strength development. This indicates an increase in strength compared to both the general sample and the sample that used untreated sludge. However, the 20% replacement sample after pretreatment, although showing lower strength than the general sample, demonstrated an improvement over the corresponding untreated sample.

Furthermore, the bending strength tests, depicted in Figure 8b, followed a similar pattern to the compressive strength results. The general sample recorded flexural strengths of 3.1 MPa after 3 days, 4.7 MPa after 7 days, and 5.5 MPa after 28 days. In contrast, the sample with a 10% replacement of untreated sludge exhibited strengths of 3.2 MPa after 3 days, 3.9 MPa after 7 days, and 3.8 MPa after 28 days, indicating a decrease in strength compared to the general sample. The strength reduction was more pronounced in the sample with a 20% sludge replacement before pretreatment. However, for the samples using sludge that had undergone the pretreatment process, the 10% replacement sample demonstrated improved flexural strengths of 3.4 MPa after 3 days, 5.0 MPa after 7 days, and 5.5 MPa after 28 days, matching the strength of the general sample by the 28th day. This improvement can be explained by the enhanced reactivity of the pretreated sludge due to the release of reactive CaO, which facilitates pozzolanic reactions [13]. In contrast, the 20% replacement sample, while showing lower strength than the general sample, exhibited an increase in strength compared to the sample using untreated sludge.

### 3.3. Water Absorption and Bond Strength Test

**Figure 9:**
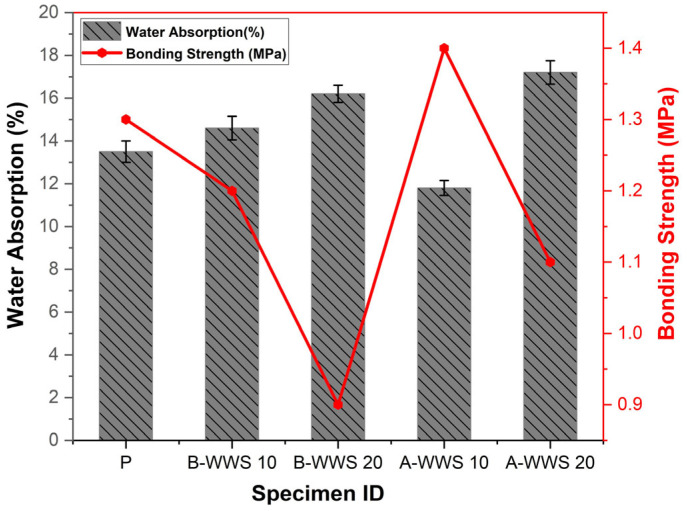
Water absorption rate.

After 28 days of curing, the water absorption test was conducted on mortar specimens, and the results are presented in Figure 9. The test results show that the control sample had a water absorption rate of 13.5%. In comparison, the sample with a 10% replacement of untreated wastewater sludge had a slightly higher rate of 14.6%, and the one with 20% replacement reached 16.2%. The higher absorption in these untreated samples can be explained by the nature of the sludge, which contains organic matter and impurities [10]. These components create gaps and microcracks in the structure, allowing water to infiltrate more easily. Without proper treatment, the sludge does not bond well with the cement matrix, leaving voids that increase water absorption. Conversely, when the sludge was pretreated, the sample with a 10% replacement showed a notably lower absorption rate of 11.8%, indicating a tighter, more compact structure. This occurs because the pretreatment process removes impurities and volatile organic compounds, making the sludge more reactive. Pretreatment also enhances bonding with the cement, filling voids and reducing spaces where water could enter, thereby creating a denser, more water-resistant material. Additionally, chemical changes from pretreatment improve the sludge’s performance in the mix. During hydration, compounds such as calcium silicate hydrates (C-S-H) and calcium aluminate hydrates (C-A-H) form, which strengthen the cement matrix and reduce porosity [15]. These compounds effectively “seal” the material, lowering water absorption. However, with a 20% replacement of pretreated sludge, the absorption rate increased to 17.2%, likely due to an imbalance in the mix, creating uneven areas and allowing more water infiltration. This finding suggests an optimal sludge replacement level, where too little yields minimal benefits, and too much weakens the structure. Furthermore, bonding strength, or the material’s ability to hold together, is greatly influenced by the hydration products formed during curing. When 10% pretreated sludge was used, the bonding strength reached 1.4 MPa, the highest in the tests. This improvement occurs because pretreated sludge enhances the pozzolanic reaction, which promotes the formation of calcium silicate hydrates (C-S-H), acting as the “glue” in cement that binds particles together, thereby strengthening the material. Pretreated sludge also reinforces the connection between the aggregate (solid particles in the mix) and the cement matrix. The finer, more reactive particles of pretreated sludge fill small gaps, creating a tighter bond. Additionally, the pozzolanic reaction with silica and alumina from the sludge produces extra binding phases like calcium alumino-silicate hydrates (C-A-S-H), further enhancing strength and durability [36]. In contrast, untreated sludge, with residual organic material and impurities, weakens bonding. Unreacted organic content creates weak points, increasing the risk of cracking and separation. Consequently, structural integrity is compromised when untreated sludge is used in larger quantities.

Furthermore, bonding strength, which is the material’s ability to hold together, is heavily influenced by the hydration products formed as the cement cures. In particular, when 10% of pretreated sludge was used, the bonding strength reached 1.4 MPa, the highest in the tests. This improvement happens because pretreated sludge enhances what is called the pozzolanic reaction. In simple terms, this reaction helps the formation of calcium silicate hydrates (C-S-H), which act as the “glue” in cement, binding everything together and giving the material its strength. Pretreated sludge also helps strengthen the connection between the aggregate (the solid particles in the mix) and the cement matrix. Because the pretreated sludge particles are finer and more reactive, they fill in the small gaps, creating a tighter bond. Additionally, the pozzolanic reaction involving silica and alumina from the sludge helps produce extra binding phases like calcium alumino-silicate hydrates (C-A-S-H), further improving the material’s strength and durability [41]. Conversely, untreated sludge, which still contains organic material and impurities, weakens the bonding. The unreacted organic content creates weak spots where the material is more prone to cracking and separating. As a result, the overall structural integrity is compromised when untreated sludge is used in higher quantities.

### 3.4. Chemical Attack Test

**Figure 10:**
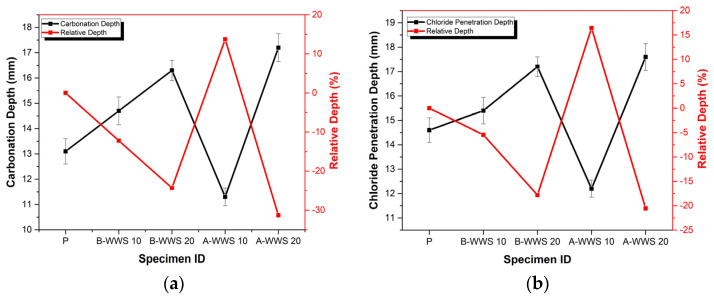
Chemical attack test, (**a**) carbonation attack, and (**b**) chloride penetration depth.

Figure 10a,b depict the carbonation depth and chloride penetration depth, respectively. The carbonation depth of the control specimen (P) was 13.1 mm, the samples with untreated sludge (B-WWS 10 and B-WWS 20) showed increased carbonation depths of 14.7 and 16.3 mm, respectively. This suggests that untreated sludge increased the matrix’s porosity, allowing for deeper CO_2_ penetration due to the presence of organic matter and non-reactive components, which created voids. In contrast, the pretreated sludge samples (A-WWS 10 and A-WWS 20) demonstrated improved carbonation resistance. The 10% pretreated sample (A-WWS 10) exhibited the lowest carbonation depth at 11.3 mm, about 14% lower than the control. Pretreatment likely removed volatile organic content, leading to a denser and less permeable matrix that limited CO_2_ ingress. Furthermore, metal oxides from the sludge may have formed protective layers on pore walls, enhancing resistance. However, the 20% pretreated sample (A-WWS 20) showed a higher carbonation depth of 17.2 mm, indicating that excessive sludge replacement disrupts the matrix, allowing more CO_2_ to penetrate. The optimal performance of the A-WWS 10 sample, both in terms of carbonation depth and relative resistance, suggests that using pretreated sludge in appropriate proportions can improve durability by reducing carbonation susceptibility.

In Figure 10b, a chloride ion penetration resistance test was carried out to evaluate the durability of cement-based materials partially replacing WWS. The control specimen had a penetration depth of 14.6 mm. In comparison, the samples with untreated sludge had higher chloride ion penetration. The 10% sludge replacement sample had a depth of 15.4 mm, and the 20% replacement sample reached 17.2 mm. This increase is likely because untreated sludge contains organic materials and other impurities that do not bond well with the cement. These unreacted components lead to microcracks and voids in the material, making it easier for chloride ions to get through [45]. On the other hand, the samples with pretreated sludge showed much better resistance to chloride penetration. The 10% pretreated sludge sample had a depth of just 12.2 mm, which is a 16% improvement compared to the control. This improvement is likely due to the pretreatment process, which removes organic matter and produces finer, more reactive particles. This leads to a denser, less porous structure, making it harder for chloride ions to pass through. Pretreated sludge also seems to help distribute the particles more evenly throughout the material, further reducing permeability [46]. Additionally, the pretreatment may enhance the presence of stable mineral phases like iron oxides (Fe_2_O_3_) and aluminum oxides (Al_2_O_3_) [3,45]. These minerals can react with chloride ions to form protective compounds, further reducing penetration. These mineral components trap chloride ions, lowering the overall depth of penetration. However, the 20% pretreated sludge sample had a penetration depth of 17.6 mm, slightly worse than the untreated equivalent. This suggests that adding too much sludge, even if it is pretreated, can disrupt the uniformity of the material, creating weak points where chloride ions can get through.

### 3.5. Freeze–Thaw Damage

**Figure 11:**
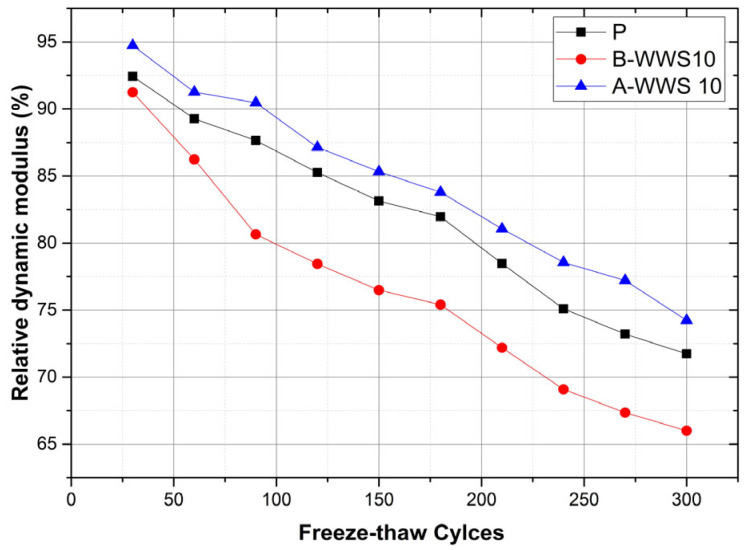
Freeze–thaw damage.

A freeze–thaw resistance test was conducted to evaluate the durability of materials containing sludge from a wastewater treatment facility against repeated freezing and thawing cycles. The results, illustrated in Figure 11, compare the performance of the control sample (P), a sample with 10% untreated sludge (B-WWS 10), and a sample with 10% pretreated sludge (A-WWS 10) over 300 cycles. In the control sample (P), resistance to freeze–thaw damage began to decrease significantly after 210 cycles, reaching 73% by the 300th cycle. The sample with 10% untreated sludge (B-WWS 10) performed worse, falling below 80% resistance after just 150 cycles and ending with only 67% resistance. The untreated sludge likely introduced more porosity into the material, allowing water to seep in. As this water freezes, it expands, causing internal damage such as microcracks, which worsened with each freeze–thaw cycle. In contrast, the sample with 10% pretreated sludge (A-WWS 10) exhibited much better performance, maintaining over 80% resistance until after 240 cycles and still retaining 75% resistance at the end of 300 cycles—outperforming both the control and untreated sludge samples. The pretreatment process likely removed impurities from the sludge, reducing its porosity and making the material more compact and resistant to water infiltration [7]. This reduced internal stresses from freezing and thawing, allowing the material to remain more durable over time. These results demonstrate that untreated sludge weakens the material’s durability by increasing its porosity and susceptibility to freeze–thaw damage, whereas pretreated sludge significantly enhances freeze–thaw resistance, as evidenced in the A-WWS 10 sample.

The ability of cement-based materials to resist freeze–thaw cycles mainly depends on how well they can limit water absorption and handle the expansion of water when it freezes. The sample with untreated sludge (B-WWS 10) did not perform well, likely because the sludge created tiny voids and capillaries in the material, which allowed it to retain more water. When water freezes, it expands by about 9%, causing internal stress that can lead to the formation of microcracks. Each freeze–thaw cycle makes these cracks worse, progressively weakening the material, as shown by the sharp drop in its relative dynamic modulus [47].

### 3.6. Microstructure Analysis

**Figure 12:**
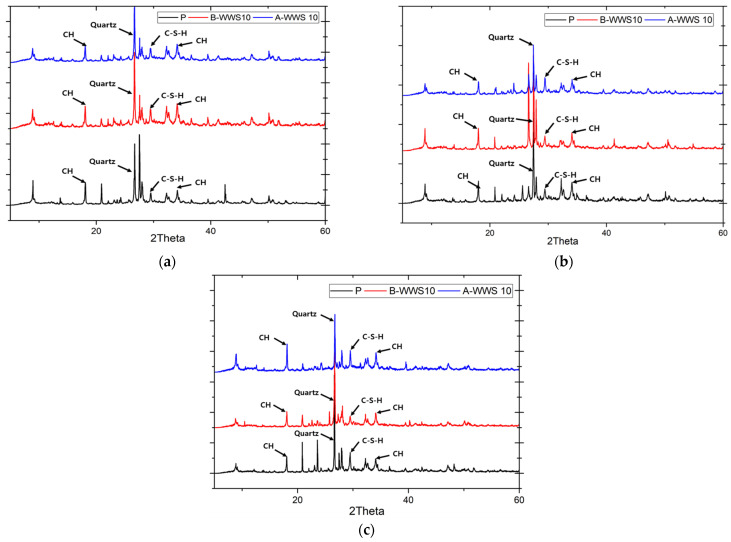
XRD spectrum of repair specimen: (**a**) @ 3 days, (**b**) @ 7 days, (**c**) @ 28 days.

XRD analysis was carried out to understand the hydration behavior of the maintenance material when partially replaced with WWS. The results, shown in Figure 12, offer a closer look at how the material developed over time. After 3 days, the XRD results revealed a higher quartz peak in the samples containing sludge compared to the control. This makes sense given that sludge often contains higher levels of silicon (Si), which likely contributed to the increased quartz content. At this early stage, the calcium hydroxide (CH) and calcium silicate hydrate (C-S-H) peaks looked fairly similar across all samples, suggesting that the initial hydration process was progressing similarly in all materials. However, after 7 days, the differences became more noticeable. The sample containing pretreated sludge (A-WWS 10) showed a much higher C-S-H peak than the other two, indicating more active hydration and formation of C-S-H, a key compound that contributes to the material’s strength [14]. This trend continued after 28 days, where the pretreated sludge sample continued to outperform the other samples, showing the most pronounced C-S-H peak. On the other hand, the sample with untreated sludge (B-WWS 10) showed a relatively lower C-S-H peak, suggesting less effective hydration. These results align well with the strength development we observed in the mechanical tests. The pretreated sludge sample, which had the highest C-S-H formation, also demonstrated better strength [48]. This makes sense, as the formation of C-S-H is closely tied to the material’s ability to gain strength over time. Meanwhile, the untreated sludge sample, which showed weaker C-S-H formation, also had lower strength results.

**Figure 13:**
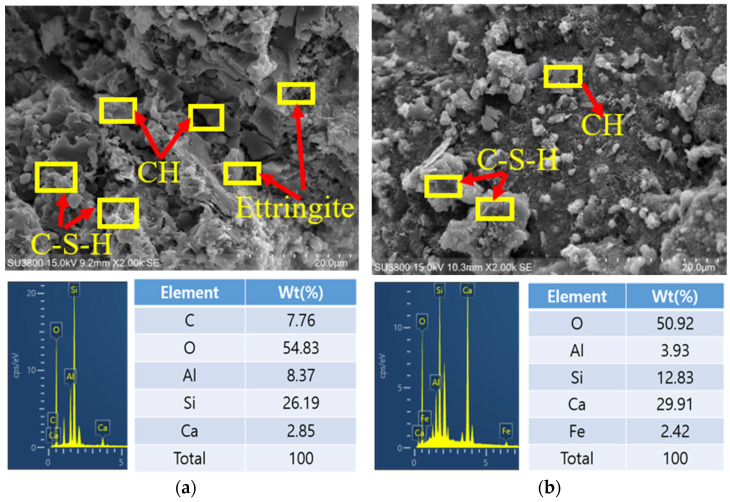

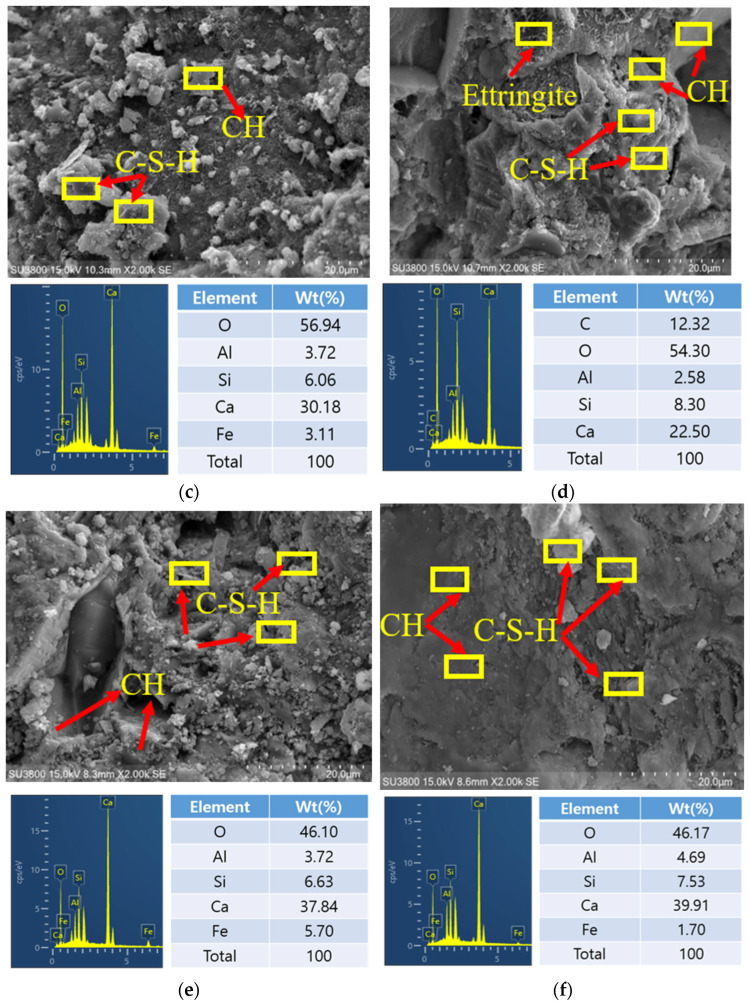
SEM analysis, (**a**) Control @ 3 days, (**b**) Control @ 28 days, (**c**) B-WSS 10 @ 3 days, (**d**) WSS 10 @ 28 days, (**e**) B-WSS @ 3 days, and (**f**) A-WSS @ 28 days.

To understand the development of hydration products in cementitious materials substituted with sludge from a sewage treatment facility, both SEM imaging and EDS analysis were performed. The results, shown in Figure 13, provide key insights into how the microstructure and hydration products evolved over time. On the third day, the SEM images showed that both the control sample and those containing sludge had started to form calcium hydroxide (CH) and ettringite, a crystalline product that forms early during the hydration process and helps the material set [7,49]. However, in the sample with untreated sludge, this process was noticeably slower. This delay could be due to impurities like organic matter and other unreactive materials in the untreated sludge, which interfere with the normal hydration reactions. By the 28th day, we saw a significant formation of calcium silicate hydrate (C-S-H), which is essential for strength, in both the control and pretreated sludge samples. The pretreated sludge sample, in particular, showed a denser and more cohesive structure, indicating that the pretreatment process played a role in accelerating hydration and improving the material’s overall quality. Pretreatment likely removes unwanted organic content from the sludge, allowing the particles to integrate more effectively with the cement, creating fewer voids and leading to a stronger, more durable material. In contrast, the sample with untreated sludge showed fewer hydration products at this stage. The lower density of C-S-H in the untreated sample reflects slower hydration, which could be attributed to the presence of unreactive particles that interfere with the formation of critical hydration compounds. This results in a more porous and weaker structure, which impacts the material’s ability to withstand long-term environmental stressors like freeze–thaw cycles and chemical exposure.

### 3.7. Environmental Hazard Analysis

To ensure that using sludge from a wastewater treatment facility in maintenance materials does not pose any environmental risks, a hazardous factor detection test was conducted following waste processing standards. The criteria and test results are presented in Table 5. The test looked for six key heavy metals: lead (Pb), arsenic (As), mercury (Hg), cadmium (Cd), copper (Cu), and hexavalent chromium (Cr^6^⁺). Fortunately, lead, arsenic, mercury, and cadmium were not detected at all, meaning their levels were so low they could not even be measured. This is a positive outcome since these metals are known for their toxicity and environmental harm. Copper and hexavalent chromium were found in very small amounts—0.020 and 0.035 mg/L, respectively, both well below the 3 and 1.5 mg/L respective safety limits for copper and hexavalent chromium. Notably, hexavalent chromium, which can be harmful even in small amounts, was detected at a level over 40 times lower than the permissible standard, confirming it is not a cause for concern. With such low levels of these heavy metals, the material is safe for long-term use without the risk of harmful chemicals leaching into the environment. Additionally, no organic phosphorus was detected, which means there is little chance of nutrient pollution or issues like algae blooms. However, for long-term safety, additional leachability tests and environmental simulations could provide a fuller picture of potential hazards. Leachability Testing Under Accelerated Aging: Aging tests using acidic and alkaline solutions could simulate extreme environmental conditions and assess the stability of heavy metals over time. Bioavailability of Heavy Metals: Long-term application of WWS mortar in open environments might involve exposure to soil and water ecosystems. Testing the bioavailability and potential uptake of heavy metals, especially chromium and copper, could further address environmental concerns.

### 3.8. Discussion on Comparison with Other Studies

The findings of this study, particularly in terms of compressive strength, flow, and final setting time, are compared with prior research conducted by Gu et al. [50], Chang et al. [18], and Garces et al. [51], as shown in Table 6. In terms of compressive strength at 28 days, this study achieved 42.1 MPa, which is slightly lower than the values reported by Gu et al. (49.3 MPa) and Garces et al. (50.3 MPa) but higher than the 38.5 MPa observed by Chang et al. Although this compressive strength is marginally lower than some previous findings, it remains within a viable range for structural applications. This difference could be attributed to variations in mixture proportions, raw materials, or curing conditions. Despite the slight reduction in strength, the material’s performance remains suitable for practical applications, especially when balanced with other desirable properties such as workability and setting time. The flow value of 170 mm in this study significantly exceeds those in the other studies, which ranged from 95 mm (Gu et al.) to 120 mm (Chang et al.). This higher flow value suggests an improved workability, likely due to optimized proportions or the inclusion of specific additives that enhance flow without compromising the material’s stability. Enhanced flowability can be advantageous in applications requiring easy placement and uniformity, especially in structures with intricate formwork or where ease of handling is prioritized. The increased workability observed in this study may help reduce labor effort and improve the efficiency of on-site applications, highlighting a practical advantage of this mix design. In terms of setting time, this study recorded a final setting time of 250 min, which is consistent with the findings of Garces et al. and notably shorter than the 400 min observed by Gu et al. A shorter setting time can be beneficial in fast-paced construction projects, as it allows for quicker formwork removal and accelerates the construction timeline. The reduced setting time observed here may also indicate a higher reactivity of the cementitious matrix, possibly due to the type of sludge or additives used, which promote faster hydration and strength gain. This accelerated setting can be an asset in time-sensitive applications, although it requires careful handling to ensure proper finishing and surface quality.

The heavy metal analysis of this study’s maintenance material demonstrates an environmentally sound composition, especially when compared with prior research findings by Mahutiane et al. [52], Chen et al. [16], Dz.U RP et al. [53], and Hazrati et al. [54] in Table 7. This study’s results show either non-detectable or significantly low levels of potentially hazardous metals, suggesting that the use of wastewater treatment sludge in this material does not elevate its environmental risk profile. Several elements known for their high toxicity and potential to bioaccumulate, such as lead, cadmium, and mercury, were either undetected or present in trace amounts well within safe regulatory limits. This contrasts favorably with some previous studies, where detectable levels of these elements were sometimes higher, suggesting a material composition here that minimizes potential leaching risks. The undetectable levels of these metals contribute to the material’s suitability for applications where long-term environmental safety is paramount, such as in maintenance projects that may come into contact with soil or groundwater over extended periods. The low concentrations of other metals, such as copper and chromium, further support the material’s safety profile. These metals, while essential in trace amounts, can become toxic in larger quantities, particularly in aquatic environments. The controlled levels found in this study indicate effective management of these elements, reducing the potential for harmful runoff or contamination over time. By maintaining such low metal concentrations, this material aligns well with environmentally conscious construction practices that prioritize minimizing ecological disruption. The absence of organic phosphorus, a known contributor to nutrient pollution, further enhances the material’s environmental appeal. Organic phosphorus is often associated with nutrient leaching, which can lead to eutrophication—a process that depletes oxygen in water bodies and disrupts aquatic ecosystems. The lack of detectable organic phosphorus in this study suggests that the material is unlikely to contribute to nutrient overload, making it suitable for use in settings near natural water sources or sensitive habitats. Collectively, these findings position the maintenance material as a sustainable and low-risk option for environmentally sensitive applications. By exhibiting minimal to non-detectable levels of hazardous heavy metals and organic pollutants, this material reflects a commitment to sustainable waste utilization and aligns with the broader goals of environmentally friendly construction. The low heavy metal content not only reduces immediate environmental risks but also supports long-term ecological health, highlighting the viability of wastewater sludge as a safe alternative component in building materials.

## 4. Conclusions

This experimental study aimed to produce repair mortar using wastewater sludge as a supplementary cementitious material. The study assessed the sludge’s usability by comparing its work performance, physical and mechanical properties, durability, and environmental hazards with traditional cement, and the following conclusions were drawn.

1. The substitution of cement with pretreated wastewater sludge (A-WWS) maintained flow characteristics comparable to the control mixture, while untreated sludge (B-WWS) reduced workability due to coarser particles. Pretreated sludge also resulted in a shorter setting time, indicating improved reactivity, whereas untreated sludge caused significant delays in setting due to its lower reactivity.
2. Pretreated sludge enhanced the compressive and flexural strength of mortar, particularly at a 10% substitution level, compared to untreated sludge. The pozzolanic reactions driven by pretreated sludge contributed to the formation of calcium silicate hydrates (C-S-H), improving strength. Higher sludge content (20%) showed diminishing returns in strength, especially with untreated sludge acting as a filler.
3. Mortars incorporating pretreated sludge demonstrated significantly lower water absorption and increased bond strength, which enhanced durability. Untreated sludge, with its higher porosity and organic content, resulted in increased water absorption and weaker bonding, making it less effective for long-term durability.
4. The pretreated sludge samples (A-WWS) exhibited superior resistance to both carbonation and chloride penetration compared to untreated sludge. The 10% pretreated sample demonstrated optimal performance, reducing CO_2_ and chloride ion ingress due to a denser, less porous matrix created by the pretreatment process.
5. The environmental hazard analysis confirmed that the use of wastewater sludge in mortar does not pose significant environmental risks, with heavy metal concentrations well below safety limits. This ensures that sludge-based repair mortars can be considered a sustainable and environmentally friendly option for structural repairs without compromising safety.

This study demonstrates that wastewater sludge (WWS) can serve as a viable supplementary cementitious material (SCM) at lower substitution levels, with a 10% replacement showing comparable strength to traditional mortar. However, limitations arise at higher substitution levels (20%), where reduced mechanical strength indicates that WWS may be best suited for non-structural applications, such as pavement blocks and partition walls, where moderate strength and environmental sustainability are prioritized. While heavy metal content was within safe limits, future research should address variability in sludge composition from different sources and investigate long-term durability under diverse environmental conditions. Additionally, given the relatively high energy requirements associated with processing WWS, efforts to enhance energy efficiency in treatment methods are needed to fully align this material’s use with sustainable construction practices. These steps are essential for optimizing WWS’s role in sustainable construction, with a focus on balancing environmental benefits and structural performance.

## Figures and Tables

**Table 1:** Chemical composition and physical properties of OPC.

Oxide Content/wt.%						Physical Characteristics.		
SiO_2_	Al_2_O	CaO	Fe_2_O_3_	MgO	K_2_O	Specific Gravity (g/cm^3^)	Surface Area (cm^2^/g)	Ig. Loss
20.8	6.3	62.0	3.2	2.9	2.1	3.15	3410	1.5

**Table 2:** Chemical composition and physical properties of WWS.

Oxide Content/wt.%						Physical Characteristics.	
SiO_2_	Al_2_O	CaO	Fe_2_O_3_	MgO	K_2_O	Specific Gravity (g/cm^3^)	Surface Area (cm^2^/g)
38.26	20.01	10.78	6.76	2.4	3.63	2.24	5684

**Table 3:** Mixture proportion of repairing cement mortar.

Mix ID	W/B (%)	Title 3	All Units Are (g)			
		Cement	WWS		Sand	WR (%)
			Treated	Non-Treated		
CS	40	700	-	-		
B-WWS 10		630	70	-		
B-WWS 20		560	140	-	1400	0.7
A-WWS 10		630	-	70		
A-WWS 20		560	-	140		

(WR) Water-reducer: wt. % of cement weight.

**Table 4:** Dimension of specimens.

Test ID	Specimen Dimension (mm^3^)	Shape
Compressive test	50 × 50 × 50	prism
Flexural test	40 × 40 × 160	prism
Freeze–thaw test	100 × 100 × 400	prism
Chlorine penetration test	Φ 100 × 50	Cylinder (disc)
Carbonation depth test	Φ 100 × 50	Cylinder (disc)
Water absorption rate test	50 × 50 × 50	Cubic

**Table 5:** Environmental hazard elements test result.

Elements	Standard	Test Result
Pb	<3 mg/L	N/D (Non-Detected)
Cu	<3 mg/L	0.02
As	<1.5 mg/L	N/D (Non-Detected)
Cd	<0.005 mg/L	N/D (Non-Detected)
Cr^6+^	<1.5 mg/L	0.035
Hg	<0.3 mg/L	N/D (Non-Detected)
organic phosphorus	<1 mg/L	N/D (Non-Detected)

**Table 6:** Physical properties comparison with previous studies.

Ref	Compressive Strength (28 Day)	Flow (mm)	Final Setting Time
Gu et al. [50]	49.3	95 mm	400
Chang et al. [18]	38.5	120 mm	N/A
Garces et al. [51]	50.3	117 mm	250
This study	42.1	170 mm	250

(N/A) not available.

**Table 7:** Total mass of heavy metal comparison with previous studies.

Ref	Pb	Cu	As	Cd	Cr	Hg	Organic Phosphorus
Mahutjane et al. [52]	0.203 mg/L	N/D	N/D	N/D	<0.009 mg/L	<0.002 mg/L	N/D
Chen et al. [16]	0.015 mg/L	0.0263 mg/L	0.013 mg/L	N/D	0.321 mg/L		N/D
Dz.U.RP et al. [53]	<0.05 mg/L	<0.5 mg/L	<0.05 mg/L	<0.005 mg/L	<5 mg/L	<0.001 mg/L	N/D
This study	N/D	0.02 mg/L	N/D	N/D	0.035 mg/L	N/D	N/D
Hazrati et al. [54]	0.015 mg/L	<0.026 mg/L	0.0059 mg/L	0.0008 mg/L	0.01 mg/L	N/D	N/D

(N/D) Not Detected.

## Data Availability

The data presented in this study are available on request from the corresponding author.
